# Prevalence, Virulence Genes, Antimicrobial Susceptibility, and Genetic Diversity of *Bacillus cereus* Isolated From Pasteurized Milk in China

**DOI:** 10.3389/fmicb.2018.00533

**Published:** 2018-03-26

**Authors:** Tiantian Gao, Yu Ding, Qingping Wu, Juan Wang, Jumei Zhang, Shubo Yu, Pengfei Yu, Chengcheng Liu, Li Kong, Zhao Feng, Moutong Chen, Shi Wu, Haiyan Zeng, Haoming Wu

**Affiliations:** ^1^University of Chinese Academy of Sciences, South China Sea Institute of Oceanology, Chinese Academy of Sciences, Guangzhou, China; ^2^State Key Laboratory of Applied Microbiology, Southern China and Guangdong Provincial Key Laboratory of Microbial Culture Collection and Application, Guangdong Open Laboratory of Applied Microbiology, Guangdong Institute of Microbiology, Guangzhou, China; ^3^Department of Food Science and Technology, Jinan University, Guangzhou, China; ^4^College of Food Science, South China Agricultural University, Guangzhou, China

**Keywords:** *Bacillus cereus*, pasteurized milk, risk assessment, virulence genes, antibiotic resistance, ERIC-PCR, genetic polymorphism, food-borne pathogen

## Abstract

*Bacillus cereus* is a common and important food-borne pathogen that can be found in various food products. Due to low-temperature sterilization for a short period of time, pasteurization is not sufficient for complete elimination of *B. cereus* in milk, thereby cause severe economic loss and food safety problems. It is therefore of paramount importance to perform risk assessment of *B. cereus* in pasteurized milk. In this study, we isolated *B. cereus* from pasteurized milk samples in different regions of China, and evaluated the contamination situation, existence of virulence genes, antibiotic resistance profile and genetic polymorphism of *B. cereus* isolates. Intriguingly, 70 samples (27%) were found to be contaminated by *B. cereus* and the average contamination level was 111 MPN/g. The distribution of virulence genes was assessed toward 10 enterotoxigenic genes (*hblA, hblC, hblD, nheA, nheB, nheC, cytK, entFM, bceT*, and *hlyII*) and one emetic gene (*cesB*). Forty five percent strains harbored enterotoxigenic genes *hblACD* and 93% isolates contained *nheABC* gene cluster. The positive rate of *cytK, entFM, bceT, hlyII*, and *cesB* genes were 73, 96, 75, 54, and 5%, respectively. Antibiotic susceptibility assessment showed that most of the isolates were resistant to β-lactam antibiotics and rifampicin, but susceptible to other antibiotics such as ciprofloxacin, gentamicin and chloramphenicol. Total multidrug-resistant population was about 34%. In addition, *B. cereus* isolates in pasteurized milk showed a high genetic diversity. In conclusion, our findings provide the first reference on the prevalence, contamination level and characteristics of *B. cereus* isolated from pasteurized milk in China, suggesting a potential high risk of *B. cereus* to public health and dairy industry.

## Introduction

The opportunistic pathogen *Bacillus cereus* is known to cause food-borne outbreaks in humans. *B. cereus* generally causes two types of gastrointestinal illness including emesis and diarrhea after consumption of a contaminated food, which contains more than 10^4^–10^5^ spores or vegetative cells of *B. cereus* per gram (Jensen et al., 2003; Bamnia and Kaul, 2015). The investigation during 1960 to 1992 had shown that food-borne outbreaks associated with *B. cereus* ranged from 1 to 22% in Europe, Japan, and North America (Griffiths and Schraft, 2002). *B. cereus* has emerged as the second food-borne pathogen in France after *Staphylococcus aureus* (Santé Publique France, 2015; Glasset et al., 2016), and has ranked third in China (Mao et al., 2010).

The pathogenicity of *B. cereus* is caused by different toxins produced by this bacterium. Diarrhea is associated with a series of enterotoxins including hemolysin BL (Hbl), non-hemolytic enterotoxin (Nhe), cytotoxin K (CytK) and enterotoxin FM (Fagerlund et al., 2004; Ehling-Schulz et al., 2006), as well as potential enterotoxins hemolysin II (HlyII) and enterotoxin T (BceT) (Agata et al., 1995a). Emetic syndrome is caused by the toxin cereulide which is synthesized by non-ribosomal peptide synthetases encoded by *ces* gene cluster (Ehling-Schulz et al., 2005, 2015). Unlike enterotoxins, cereulide is a thermo- and acidic-stable depsipeptide (Rajkovic et al., 2008) that is preformed in contaminated foods. Moreover, *B. cereus* involves in many serious and potentially fatal non-gastrointestinal-tract infections such as severe eye infections, osteomyelitis, hepatitis and inflammatory responses (Bottone, 2010; Rishi et al., 2013), and even death (Lund et al., 2000; Posfay-Barbe et al., 2008).

Antibiotic therapy is still the primary treatment method for the infections of *B. cereus*. However, emergence of antibiotic resistant *B. cereus* strains, mainly due to antibiotic misusage (Barbosa and Levy, 2000) or acquisition of resistance genes through horizontal gene transfer (Bogdanova et al., 1998; Agerso et al., 2002; Brown et al., 2003), results in the failure of antibiotic treatment. Thus, obtaining the *B. cereus* antibiotics resistance profile is highly relevant to public health.

*Bacillus cereus* has been found in milk with a high contamination rate (Rather et al., 2011; Gundogan and Avci, 2014; Chaves et al., 2017; Owusu-Kwarteng et al., 2017; Saleh-Lakha et al., 2017; Lan et al., 2018), especially pasteurized milk using low-temperature sterilizing process that cannot fully eliminate *B. cereus* spores (Zwietering et al., 1996). Consequently, it could lead to a variety of milk defects and foodborne diseases (Stenfors Arnesen et al., 2008). Besides, *B. cereus* in milk is probably associated with a higher risk of hazard. For example, thermophilic strains of *B. cereus* from milk had higher toxicity (Miller et al., 2016) and milk-originated *B. cereus* sensu lato strains harbored *Bacillus anthracis*-like plasmids, suggesting that *B. cereus* could gain more pathogenic genes through the interflow among *Bacillus* groups in milk (Bartoszewicz and Marjańska, 2017).

*B. cereus* contamination in dairy products have been reported (Gundogan and Avci, 2014; Yobouet et al., 2014; Drean et al., 2015; Chaves et al., 2017; Owusu-Kwarteng et al., 2017; Saleh-Lakha et al., 2017; Lan et al., 2018); however, the overall scale, especially in pasteurized milk, is quite small. Since pasteurization has a low inactivation rate of *B. cereus* spores and detection of *B. cereus* is not required for the dairy microorganism test of Chinese food security standard (The Hygiene Ministry of China, 2010b), it may increase the risk of *B. cereus* in dairy products. Thus, it is necessary to assess the prevalence and microbiological traits of *B. cereus* in pasteurized milk products. Here we used enterobacterial repetitive intergenic consensus sequences polymerase chain reaction (ERIC-PCR) to analyze genotypic diversity of *B. cereus* isolated from pasteurized milk products in China and combined with the pathogenic potential, antimicrobial resistance characters, aiming to provide an overview of the risk assessment for *B. cereus* isolated from pasteurized milk in China.

## Materials and Methods

### Sample Collection

From July 2011 to May 2016, two hundred and seventy six pasteurized milk samples were collected from major cities in China (**Supplementary Figure S1**). The investigation process was divided into three stages. The first-stage investigation mainly focused on cites in southern china, and the second stage included six cities in northern China. The third one was a national wide survey that included other 15 cities. Details of sample distribution were shown in **Table 1**.

**Table 1:** Occurrence of *Bacillus cereus* from pasteurized milk samples in China.

	Survey regions	No. of samples	Contamination rate (%)^a^	Average contamination levels (MPN/g)^b^
1	Cities in southern China^c^	132	25	149
2	Cities in northern China^c^	36	31	208
3	Other 15 cities^c^	90	29	22
	Total	258	27	111

*^*a*^Contamination rate = Number of positive samples/Total samples; ^*b*^Average contamination levels (MPN/g) = Sum of MPN values/weight of positive samples; ^*c*^Cities in southern China included Nanchang, Chengdu, Hefei, Wuhan, Shanghai, Kunming, Fuzhou, Nanning, Beihai, Xiamen, Haikou, Sanya, Shenzhen et al.; Cities in northern China consisted of Xi’an, Harbin, Jinan, Beijing, Taiyuan, Lanzhou; Other 15 cities included Changsha, Hangzhou, Guiyang, Changchun, Xining, Yinchuan, Hohhot, Shenyang, Nanjing, Macao, Shijiazhuang, Zhengzhou, Lhasa, Urumqi and Hong Kong. There were no overlapping regions between three surveys.*

### Qualitative and Quantitative Detection of *B. cereus*

The qualitative and quantitative detection of *B. cereus* were performed according to National Food Safety Standard (The Hygiene Ministry of China, 2010a) with minor modification. In brief, 25 ml of sample was mixed and homogenized with 225 ml Trypticase-soy-polymyxin (TSB) broth (Huankai, China) at 30°C for 48 h. Then cultures were streaked on the Mannitol-egg yolk-polymyxin (MYP) agar plate (Selective media; Huankai, China) and Chromogenic plate (Huankai, China) and incubated at 30°C for 24 h. Colonies with pink sparkle in blue or blue-green precipitation on Chromogenic plate were picked for further biochemical identification using the *B. cereus* biochemistry assessor (Huankai, China). The quantitative detection assay was conducted by *B. cereus* most probable number (MPN) counting method in Food Safety Standards (The Hygiene Ministry of China, 2010a).

### Detection of Virulence Genes

The genomic DNA of various strains isolated from pasteurized milk samples was extracted using the HiPure Bacterial DNA Kit (Magene, United States) in accordance with the manufacturer’s instruction.

PCR screening was employed to detect the presence of seven enterotoxigenic genes (*hblA, hblC, hblD, nheA, nheB, nheC, cytK*), three potential virulence genes (*bceT, entFM, hlyII*) and one cereulide synthetase gene (*cesB*). The PCR reaction mixture (25 μl) consisted of 50 ng genomic DNA, 2 μM primers, and 12.5 μl PCR Premix TaqTM (Takara, China). The amplification was performed as described previously (Asano et al., 1997; Hansen and Hendriksen, 2001; Fagerlund et al., 2004; Ehling-Schulz et al., 2005; Oltuszak-Walczak and Walczak, 2013). The detailed sequence information and annealing temperature of these primer sets were shown in **Table 2**.

**Table 2:** Primers used in this study.

Primer	Sequence (5′-3′)	Target fragment length (bp)	Annealing temperature (°C)	Reference
HblA-F	GTGCAGATGTTGATGCCGAT	320	55	Hansen and Hendriksen, 2001
HblA-R	ATGCCACTGCGTGGACATAT			
HblC-F	AATGGTCATCGGAACTCTAT	750	55	Hansen and Hendriksen, 2001
HblC-R	CTCGCTGTTCTGCTGTTAAT			
HblD-F	AATCAAGAGCTGTCACGAAT	430	55	Hansen and Hendriksen, 2001
HblD-R	CACCAATTGACCATGCTAAT			
NheA-F	TACGCTAAGGAGGGGCA	500	55	Hansen and Hendriksen, 2001
NheA-R	GTTTTTATTGCTTCATCGGCT			
NheB-F	CTATCAGCACTTATGGCAG	770	55	Hansen and Hendriksen, 2001
NheB-R	ACTCCTAGCGGTGTTCC			
NheC-F	CGGTAGTGATTGCTGGG	583	55	Hansen and Hendriksen, 2001
NheC-R	CAGCATTCGTACTTGCCAA			
BceT-F	CGTATCGGTCGTTCACTCGG	661	55	Hansen and Hendriksen, 2001
BceT-R	GTTGATTTTCCGTAGCCTGGG			
CytK-F	AAAATGTTTAGCATTATCCGCTGT	238	55	Oltuszak-Walczak and Walczak, 2013
CytK-R	ACCAGTTGTATTAATAACGGCAATC			
Ces-F	GGTGACACATTATCATATAAGGTG	1271	58	Ehling-Schulz et al., 2005
Ces-R	GTAAGCGAACCTGTCTGTAACAACA			
Hly II-F	GATTCTAAAGGAACTGTAG	867	50	Fagerlund et al., 2004
Hly II-R	GGTTATCAAGAGTAACTTG			
EntFM-F	ATGAAAAAAGTAATTTGCAGG	1269	60	Asano et al., 1997
EntFM-R	TTAGTATGCTTTTGTGTAACC			
ERIC -F	ATGTAAGCTCCTGGGGATTCAC	200 up	45	Versalovic et al., 1991
ERIC-R	AAGTAAGTGACTGGGGTGAGCG			

### Antimicrobial Susceptibility Testing

Antimicrobial susceptibility of all *B. cereus* isolates was evaluated by the Kirbye–Bauer disk diffusion method according to performance standards for antimicrobial susceptibility testing of the Clinical and Laboratory Standards Institute (The Clinical, and Laboratory Standards Institute [CLSI], 2010/2015) for *Staphylococcus aureus*. Twenty one antibiotics (Oxoid, United Kingdom) were tested, including ampicillin (AMP, 10 μg), amoxicillin-clavulanic acid (AMC, 20 μg/10 μg), penicillin (P, 10 U), trimethoprim-sulfamethoxazole (SXT, 1.25 μg/23.75 μg), cephalothin (KF, 30 μg), cefoxitin (FOX, 30 μg), cefotetan (CTT, 30 μg), imipenem (IPM, 10 μg), gentamicin (CN, 10 μg), kanamycin (K, 30 μg), erythromycin (E, 15 μg), telithromycin (TEL, 15 μg), vancomycin (VA, 30 μg), teicoplanin (TEC, 30 μg), ciprofloxacin (CIP, 5 μg), chloramphenicol (C, 30 μg), tetracycline (TE, 30 μg), clindamycin (DA, 2 μg), rifampin (RD, 5 μg), quinupristin (QD, 15 μg), and nitrofurantoin (FD, 300 μg). After incubating for 24 h at 37°C, the inhibition zones were measured and interpreted referring to the zone diameter interpretive criteria of *S. aureus* in **Supplementary Table S1**.

### Genetic Biodiversity Assay

The ERIC-PCR method was performed for typing and comparing the pasteurized milk isolates. ERIC–PCR was carried out using the primers of ERIC-F and ERIC-R (**Table 1**) as described by Versalovic et al. (1991). The PCR reaction mixture (25 μl) contained 12.5 μl of ExTaq Mix (Takara, China), 1.0 μM of each primer, 50–100 ng genomic DNA. Amplification was performed as follows: an initial denaturation at 94°C for 3 min; 35 cycles each consists of 30 s at 94°C, 40 s at 45°C and 3 min at 72°C; and a final extension at 72°C for 10 min. Thereafter the amplicons (10 μl) were electrophoresed on 1.5% agarose gel. The DNA fingerprint was analyzed by BioNumerics 7.1 software (Applied Maths, Belgium). ERIC-PCR cluster analysis was assessed by Simpson’s diversity index (Hunter and Gaston, 1988).

## Results

### Prevalence Analysis of *Bacillus cereus* in Pasteurized Milk

*Bacillus cereus* contamination was found to occur in pasteurized milk samples from 32 out of 39 cities in China (**Supplementary Figure S1**). Of 258 milk samples evaluated, 70 (27%) samples contained *B. cereus*. Intriguingly, the overall contamination level of *B. cereus* is significantly higher (111 MPN/g), indicating that *B. cereus* risk in pasteurized milk is serious. According to the sample collection sites, the contamination of *B. cereus* was 31% (11/36) in northern China and 25% (33/132) in southern China (**Table 1**). Among different sample collection cities, contamination is quite serious in Guangzhou, Nanjing, Xining and Shenzhen. In contrast, no contamination was detected in Shantou, Sanya, Nanning, Shanghai, Nanchang, Xi’an, and Lhasa (**Supplementary Figure S1**).

### Distribution of Virulence Genes Among *B. cereus* Isolates

**FIGURE 1:**
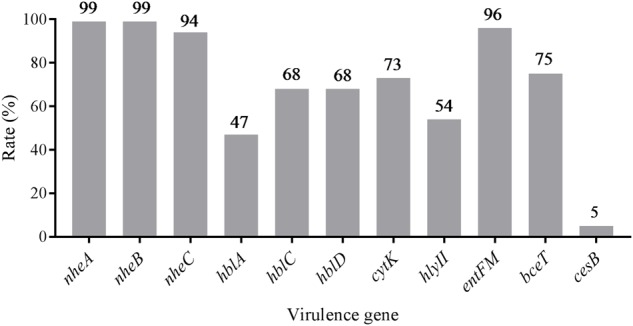
Distribution of virulence genes in *Bacillus cereus* isolated from pasteurized milk in China.

The distribution of virulence genes was evaluated and summarized in **Figure 1** and **Supplementary Table S2**. According to the pathogenic characteristics of *B. cereus*, the virulence genes are divided into two categories, namely enterotoxin genes (*nheABC, hblACD, cytK, bceT, hly II, entFM*) and cereulide synthetase genes (*cesB*). In enterotoxin genes, the gene cluster *nheABC* encoding the non-hemolytic enterotoxin (Nhe) complexes present in most of the strains (93%) with only a small portion of strains missing *nheC* or *nheA*. Forty five percent strains harbored enterotoxigenic genes *hblACD*. But the detection rate of *hblA* (46%) was significantly lower than *hblC* (66%) and *hblD* (67%). *CytK, entFM* and *bceT* were detected in more than 70% of the strains. In contrast, the detection rate of cereulide synthetase gene *cesB* was found to be only 5%, demonstrating that diarrheal strains in pasteurized milk samples are more common than emetic strains.

**FIGURE 2:**
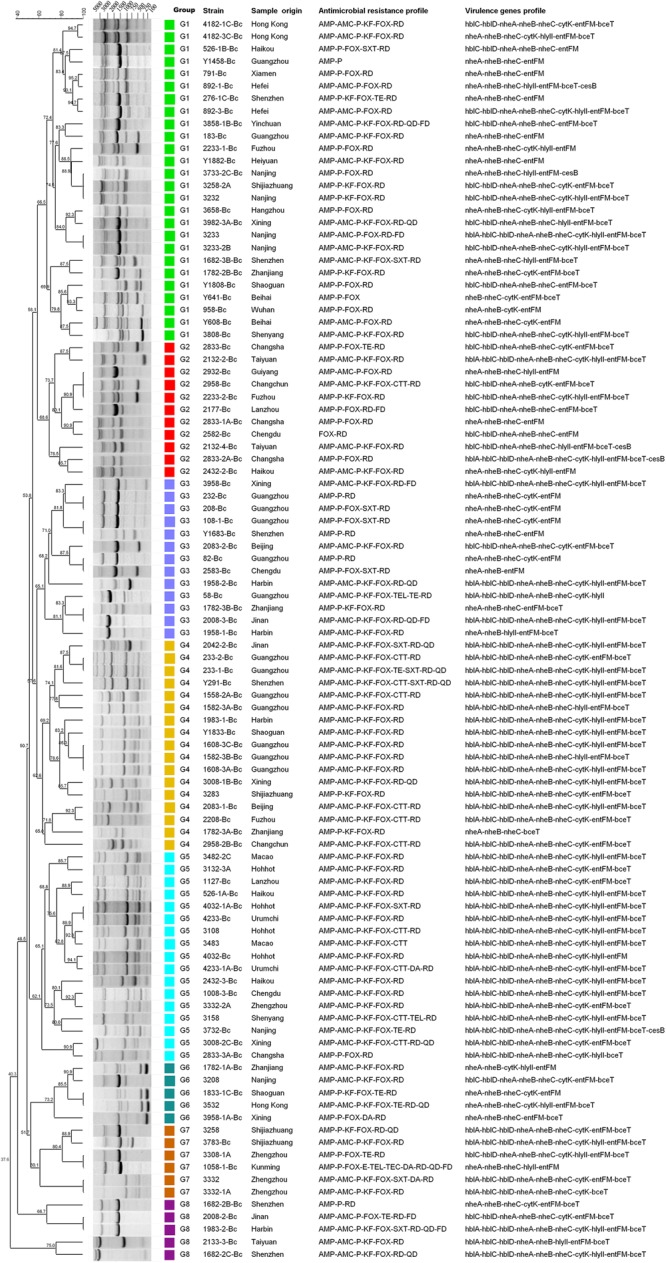
Dendrogram of *Bacillus cereus* isolated from pasteurized milk in China. Similarity (%) between fingerprints generated by ERIC-PCR was calculated by using the Dice index. The data were sorted by the UPGMA method. Different colors represent different groups, and the virulence genes profile and antimicrobial resistance characteristics of all isolates are listed in this dendrogram.

Based on the distribution of virulence genes, all isolates were divided into 32 virulence genes profiles. As shown in **Figure 2**, only two isolates (2833-2A-Bc in G2 and 3732-Bc in G5) contained all 11 virulence genes, and one isolate harbored the least virulence gene profile (2583-Bc in G3, *nheA-nheB-entFM*). The main gene profile was *hblA-hblC-hblD-nheA-nheB-nheC-cytK-hlyII-entFM-bceT* (28%), revealing that a large number of potentially diarrheal strains exist in the collected samples.

### Antimicrobial Susceptibility Tests of *B. cereus* Isolates

**FIGURE 3:**
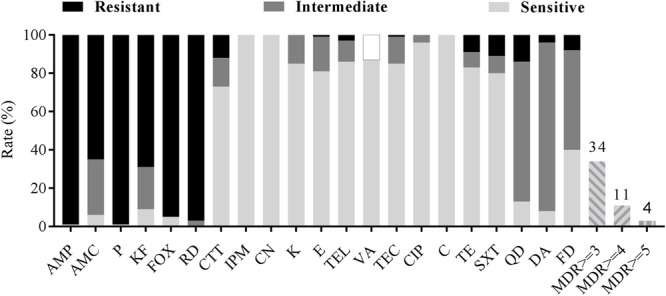
Antibiotic susceptibility of *Bacillus cereus* isolated from pasteurized milk in China. Blank bar, dark gray bar and light gray bar represent the proportion of resistant strains, moderately resistant strains and sensitive strains respectively. Gray stripe bars represent the proportion of multidrug resistance (MDR) strains. Vancomycin (VA) tested by Kirbye–Bauer disk diffusion method could only identify sensitive strains, so the black circle represents the percentage of remaining resistant and intermediate ones.

All *B. cereus* isolates were tested for antimicrobial susceptibilities to 21 selected antibiotics. As shown in **Table 3** and **Figure 3**, most of the isolates were resistant to ampicillin (AMP; 99%), penicillin (P; 99%), cefoxitin (FOX; 95%), amoxicillin-clavulanic acid (AMC; 65%) and cephalothin (KF; 69%), which belong to β-lactams. Besides, rifampin (RD) had no effect on most strains (97%). Nearly all isolates were sensitive to the remaining antibiotics, such as imipenem (IPM; 100%), gentamicin (CN; 100%), kanamycin (K; 85%), telithromycin (TEL; 86%), teicoplanin (TEC; 85%), ciprofloxacin (CIP; 96%), chloramphenicol (C; 100%) et al. In addition, most of the isolates were moderately resistant to clindamycin (DA; 88%), quinupristin (QD; 73%) and nitrofurantoin (FD; 52%). On the other hand, we also found part of isolates (13%) was not sensitive to vancomycin (VA) according to the standard of The Clinical, and Laboratory Standards Institute [CLSI] (2010/2015) for Kirbye–Bauer disk diffusion method.

**Table 3:** Results of antimicrobial resistance of *Bacillus cereus* isolates in the study.

Category	Antimicrobial agents	*Bacillus cereus* (*n* = 103)		
		Sensitive	Intermediate	Resistant
I	β-Lactams			
	Ampicillin (10 μg)	1 (1%)	0 (0%)	102 (99%)
	Amoxicillin-clavulanic acid (20 μg/10 μg)	6 (6%)	30 (29%)	67 (65%)
	Penicillin (10 units)	1 (1%)	0 (0%)	102 (99%)
	Cephalothin (30 μg)	9 (9%)	23 (22%)	71 (69%)
	Cefoxitin (30 μg)	5 (5%)	0 (0%)	98 (95%)
	Cefotetan (30 μg)	76 (74%)	15 (15%)	12 (12%)
II	Carbapenems			
	Imipenem (10 μg)	103 (100%)	0 (0%)	0 (0%)
III	Aminoglycosides			
	Gentamicin (10 μg)	103 (100%)	0 (0%)	0 (0%)
	Kanamycin (30 μg)	88 (85%)	15 (15%)	0 (0%)
IV	Macrolides			
	Erythromycin (15 μg)	83 (81%)	19 (18%)	1 (1%)
	Telithromycin (15 μg)	89 (86%)	11 (11%)	3 (3%)
V	Glycopeptides			
	Vancomycin (30 μg)	90 (87 %)	–	–
	Teicoplanin (30 μg)	88 (85%)	14 (14%)	1 (1%)
VI	Quinolones			
	Ciprofloxacin (5 μg)	99 (96%)	4 (4%)	0 (0%)
VII	Amphenicols			
	Chloramphenicol (30 μg)	103 (100%)	0 (0%)	0 (0%)
VIII	Tetracyclines			
	Tetracycline (30 μg)	86 (84%)	8 (8%)	9 (9%)
IX	Folic acid inhibitors			
	Trimethoprim-Sulfamethoxazole (1.25μg/23.75 μg)	83 (81%)	9 (9%)	11 (11%)
X	Lincosamides			
	Clindamycin (2 μg)	8 (8%)	91 (88%)	4 (4%)
The others	Rifampin (5 μg)	0 (0%)	3 (3%)	100 (97%)
	Quinupristin (15 μg)	14 (14%)	75 (73%)	14 (14%)
	Nitrofurantoin (300 μg)	42 (41%)	53 (52%)	8 (8%)
Pansusceptible				
	≥3 Antimicrobia	34%		
	≥4 Antimicrobia	11%		
	≥5 Antimicrobia	4%		

There were 34 antimicrobial resistance profiles for all isolates. As shown in **Figure 2**, the most susceptible strain resisted to only two antibiotics, while the most resistant strain resisted to ten antibiotics. AMP-AMC-P-KF-FOX-RD was the most common antimicrobial resistance profile. Multidrug resistance (MDR) profiles, defined as simultaneously resistant to more than three types of antibiotics (Magiorakos et al., 2012), were also evaluated. Thirty four percent of all isolates displayed resistance to three or more antibiotics, and 4% of the isolates displayed resistance to over five types of antibiotics (**Table 3** and **Figure 3**).

### ERIC-PCR Typing and Cluster Analysis

Molecular typing is a good way for tracing the sources and understanding the epidemiology of food-borne pathogens, such as random amplification of polymorphic DNA (RAPD, Nilsson et al., 1998), multi-locus sequence typing (MLST, Helgason et al., 2004), amplified fragment length polymorphism (AFLP, Hill et al., 2004), pulse-field gel electrophoresis (PFGE, Merzougui et al., 2014) and so on. Among these methods, ERIC-PCR (Shangkuan et al., 2000) is a simple and efficient approach to study the genetic diversity among strains that can explain the association of phenotypic and genotypic characters quite well. For our samples, we explored ERIC-PCR for genetic diversity analysis. The fingerprints gained by ERIC-PCR consisted of 2–11 distinct bands ranging in size from 250 base pairs to 5000 base pairs. Together with the distribution of virulence genes and antimicrobial susceptibility, the dendrogram was generated by using the software BioNumerics 7.0 (**Figure 2**).

All isolates showed 83 ERIC-PCR patterns. As shown in the **Figure 2**, when the relative similarity coefficient is 65%, 103 isolates could be divided into 8 clusters (G1 to G8). The G1 strains were dominant in our national isolates of which the fingerprints consisted of 6 to 8 bands and shared the common bands in sizes of about 1500 base pairs, 3000 base pairs and 4000 base pairs. As a discriminatory index, Simpson’s diversity is used to evaluate different typing methods (de la Puente-Redondo et al., 2000; Almeida et al., 2016; Wasfi et al., 2016), which produces values in the ranges of 0.0–1.0. The value 1.0 indicates that a typing method is able to distinguish each member of a population. Conversely, 0.0 indicates that all members of a population are of an identical type (Hunter and Gaston, 1988). If Simpson’s index (DI value) of a typing method is greater than 0.90, it is suggested that the typing method has generated a good result to distinguish all isolates. In our study, the DI value was 0.996, indicating the ERIC-PCR typing method could discriminate all the isolates well. Moreover, G1 (25.2%), G4 (16.5%), and G5 (16.5%) were the dominant groups. The fingerprints of G4 and G5 were quite similar, as well as their drug resistance spectra and virulence gene profiles. These two groups accounted for 41% of all strains, with *hblA/C/D-nheA/B/C-cytK-hlyII-entFM-bceT* as the main virulence gene type and AMP-AMC-P-KF-FOX-RD as the main drug resistance spectrum, indicating that the genetic diversity of these isolates were somehow conservative.

## Discussion

### The Prevalence and Genetic Diversity of *B. cereus* Isolates

Till now, the prevalence studies of pathogenic *B. cereus* in pasteurized milk in China are scant. In this study, *B. cereus* was detected in 27% of pasteurized milk samples collected from major cities in China, suggesting a potential risk of *B. cereus*. Compared to previous surveys in other countries, it is a medium contamination level compared with 27% in Abidjan (Yobouet et al., 2014), 27.37% in Brazil (Reis et al., 2013), 14% in Slovakia (Acai et al., 2014), 47% in Ghana (Owusu-Kwarteng et al., 2017) and 37% in India (Rather et al., 2011). Traced to the source of *B. cereus* contamination in pasteurized milk, heat stable *B. cereus* spores in raw milk and the post-pasteurization contamination along the milk processing lines were major sources (Lin et al., 1998; Saleh-Lakha et al., 2017). Besides, the environments for milk production, handling and processing could introduce *B. cereus* into dairy products (Cui et al., 2016a; Kumari and Sarkar, 2016). Some studies reported the storage temperature of dairy products also affected the number and toxicity of *B. cereus* (Fermanian et al., 1997; Notermans et al., 1997) as toxic strains could produce toxin even at 8°C. Together, these analyses imply the high prevalence of *B. cereu*s and existence of potential hazards in consuming of the contaminated pasteurized milk.

The epidemiological typing is considered a crucial tool for studying the prevalence of food-borne bacteria. ERIC-PCR is based on the targeting of repeated DNA sequences with oligonucleotide primers, which has been broadly employed to perform the epidemiological typing of micro-organisms and widely applied into the risk survey of pathogenic bacteria such as *Salmonella Typhimurium* (Almeida et al., 2016), *Staphylococcus aureus* (Miao et al., 2016), *Klebsiella pneumonia* (Wasfi et al., 2016) and *Arcobacter* spp. (Vicente-Martins et al., 2018) as well as *B. cereus* strains (Hsueh et al., 1999; Shangkuan et al., 2000; Lopez and Alippi, 2007; Freitas et al., 2008; Avsar et al., 2017). In this study, we used ERIC-PCR to analyze the genetic and biological diversity of *B. cereus* isolates from pasteurized milk in China. The results showed that ERIC typing was suitable for studying the relationship between genetic and biological characteristics (**Figure 2**). The dominant genotype defined by ERIC fingerprints had the main virulence gene profile *hblA/C/D-nheA/B/C-cytK-hlyII-entFM-bceT* and drug resistance spectrum AMP-AMC-P-KF-FOX-RD, indicating the correlation between these different traits. Previous studies showed that fingerprinting patterns were used to distinguish different species such as AFLP typing in grouping *B. anthracis, B. cereus*, and *B. thuringiensis* isolates (Hill et al., 2004) and RAPD typing for discrimination of the *B. cereus* groups (Kuwana et al., 2012). However, there are very few studies available on fingerprint typing to correlate the genetic characteristics with biological traits of different strains. Here, in this study, we found that ERIC-PCR had great advantages in distinguishing and classifying strains with different biological characteristics.

### Virulence Genes of *B. cereus* Isolates

Unlike emesis, diarrhea is associated with a series of enterotoxins produced by *B. cereus* in the small intestine, and the predominant protein responsible for this has not been yet determined other than enterotoxins (Jessberger et al., 2015). Nevertheless, pore-forming cytotoxins haemolysin BL (Hbl), non-haemolytic enterotoxin (Nhe) and cytotoxin K (CytK) have been identified as etiological agents of the diarrheal disease (Lund and Granum, 1996; Lund and Granum, 1999; Lund et al., 2000). In this study, 10 enterotoxigenic genes were detected and the proportion of *nheABC, hblACD* and *cytK* in *B. cereus* were found to be 93, 45, and 73%, respectively, which is quite similar to previous studies in France (*nhe* 96%, *hbl* 40%, *cytk* 42%, Glasset et al., 2016) or in China (*nhe* 100%, *hbl* 78.3%, Cui et al., 2016b), but were higher than an investigation of dairy products in Turkey (*hbl* 13%, *nhe* 60%, *cytk* 75%, Yibar et al., 2017), indicating that diarrheal strains have a wider distribution and a higher risk of *B. cereus* infections exists in consuming pasteurized milk in China.

Emetic symptom is triggered by the heat-stable dodecadepsipeptide cereulide, and a minimal emesis-causing dose was reported to be 8–10 μg/kg body weight in animal experiments (Agata et al., 1995b; Shinagawa et al., 1995). The emetic disease has often been connected with the consumption of fried and cooked rice (Agata et al., 2002; Kim et al., 2017), or pasta, pastry and noodles (Schoeni and Wong, 2005). In dairy products, strains with emetic toxin encoding genes were rare (Svensson et al., 2006; Saleh-Lakha et al., 2017). In our samples, the emetic strain was identified to be about 5%, which is not comparable to recent studies (10.2%, Biesta-Peters et al., 2016; 9%, Owusu-Kwarteng et al., 2017), but is much higher than others (1.0–3.8%, Svensson et al., 2006; 2%, Chaabouni et al., 2015; 1.1%, Cui et al., 2016a). According to previous study, strains isolated from dairy products present strong toxicity (7–15.3 folds higher than the reference emetic strain; Cui et al., 2016b). Congruently, earlier report showed that emetic strains could produce cereulide even at low temperature (Thorsen et al., 2006). So the emetic *B. cereus* is also a potential risk in pasteurized milk.

### Antimicrobial Susceptibility of *B. cereus*

*Bacillus cereus* may cause severe diseases and infections that even lead to death (Lund et al., 2000; Dierick et al., 2005). Effective antibiotic therapy is considered as a predominant treatment to eliminate *B. cereus* infections, which has necessitated the investigation of antimicrobial susceptibility of *B. cereus*. In our study, *B. cereus* isolates were resistant to β-lactam antibiotics and rifampicin, but were susceptible to quinolones, aminoglycosides and macrolides. Consistent with previous studies, *B. cereus* were resistant to β-lactam antibiotics (Luna et al., 2007; Fernandes et al., 2014; Kim et al., 2015; Yibar et al., 2017) owing to the β-lactamase production (Bottone, 2010). It is worth mentioning that *B. cereus* was resistant to cephalosporin (cefoxitin, cephalothin) except for the third generation cephalosporin (cefotetan). Since broad-spectrum cephalosporin is the main antibiotic used in the treatment of gastrointestinal diseases caused by bacterial infections, it should be avoided as clinical treatment to gastroenteritis caused by *B. cereus* (Savic et al., 2016). Nowadays, vancomycin is considered as one of the most proper choice for *B. cereus* infections (Tatara et al., 2013; Torkar et al., 2016). However, a portion of our isolates (about 13%) detected were not sensitive to this antibiotic, suggesting the existence of potential risk for *B. cereus* infections. For multiple drug resistance, *B. cereus* isolates displayed resistance to three or more antibiotics were 34% which should be paid more attention to.

## Conclusion

Despite the potential health risks associated with *B. cereus*, the prevalence and molecular analysis of genotypes of them have not been fully explored in pasteurized milk in China. In this study, we showed the high prevalence of *B. cereus* and its antibiotic resistance characteristics in pasteurized milk all over China. ERIC-PCR analysis demonstrated high genetic diversity of *B. cereus* in pasteurized milk samples. To the best of our knowledge, this is the first comprehensive investigation about the prevalence of *B. cereus* virulence factors, antibiotic resistance phenotypes and genotypes by ERIC typing in pasteurized milk from diverse regions of China that revealed a potential high risk of *B. cereus* to public health and dairy industry.

## Author Contributions

QW, YD, JW, JZ, and TG conceived the project and designed the experiments. TG, SY, PY, CL, LK, ZF, MC, SW, HZ, and HW performed the experiments. QW and YD supervised the project. TG and YD analyzed the data and wrote the article. QW, JW, and YD complemented the writing.

## Conflict of Interest Statement

The authors declare that the research was conducted in the absence of any commercial or financial relationships that could be construed as a potential conflict of interest.

## Supplementary Material

FIGURE S1 Map of China showing the cities where the *Bacillus cereus* isolates were collected.

TABLE S1 Zone diameter interpretive criteria in this study.

TABLE S2 Prevalence of virulence genes in *Bacillus cereus* isolated from pasteurized milk in China.
