# Supplementary figures and images for: Prevalence, Virulence Genes, Antimicrobial Susceptibility, and Genetic Diversity of Bacillus cereus Isolated From Pasteurized Milk in China
Source: Front Microbiol. 2018 Mar 26;9:533. doi: 10.3389/fmicb.2018.00533 (PMC5879084; doi:10.3389/fmicb.2018.00533)

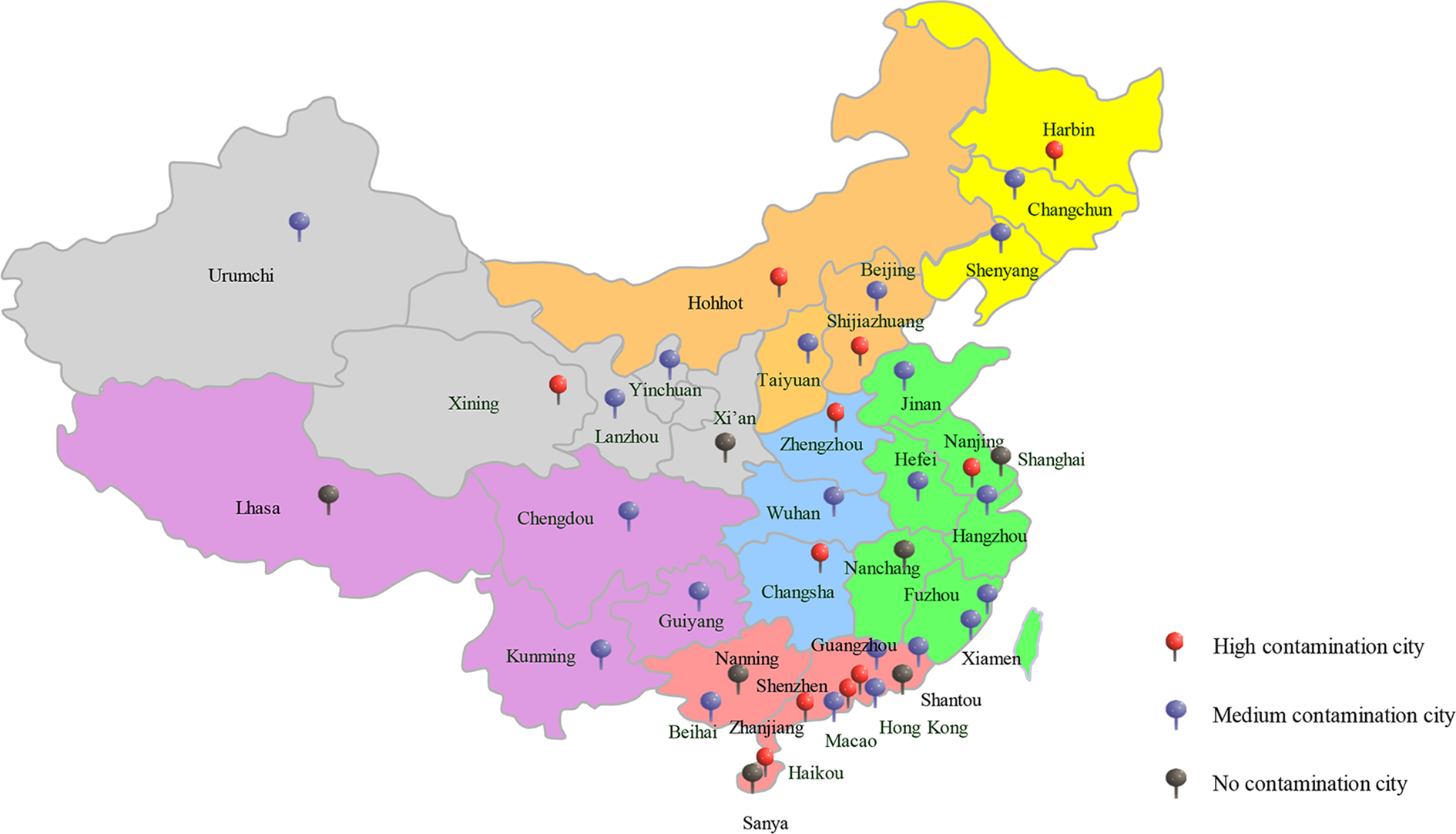

Supplement: FIGURE S1 — Map of China showing the cities where the Bacillus cereus isolates were collected. [file Image_1.TIF]
