# Supplementary material for: Prevalence, Virulence Genes, Antimicrobial Susceptibility, and Genetic Diversity of Bacillus cereus Isolated From Pasteurized Milk in China
Source: Front Microbiol. 2018 Mar 26;9:533. doi: 10.3389/fmicb.2018.00533 (PMC5879084; doi:10.3389/fmicb.2018.00533)
Supplement: TABLE S1 — Zone diameter interpretive criteria in this study. [file Table_1.DOCX]

**Supplementary Table 1 Zone diameter interpretive criteria in this study.**

| **Antibiotic type** | **Antibiotic** | **Zone Diameter Criteria (mm)** | |
| --- | --- | --- | --- |
|  |  | **R (≤)** | **S (≥)** |
| β-lactam antibiotics | Ampicillin | 28 | 29 |
|  | Amoxicillin | 13 | 18 |
|  | Penicillin | 28 | 29 |
|  | Cephalothin | 14 | 22 |
|  | Cefoxitin | 21 | 22 |
|  | Cefotetan | 12 | 16 |
| Carbapenems | Imipenem | 13 | 16 |
| Aminoglycosides | Gentamicin | 12 | 15 |
|  | Kanamycin | 13 | 18 |
| Macrolides | Erythromycin | 13 | 23 |
|  | Telithromycin | 18 | 22 |
| Glycopeptides | Vancomycin | -- | 15 |
|  | Teicoplanin | 10 | 14 |
| Quinolones | Ciprofloxacin | 15 | 21 |
| Amphenicols | Chloramphenicol | 12 | 18 |
| Tetracyclines | Tetracycline | 14 | 19 |
| Folic acid inhibitors | Sulfamethoxazole | 10 | 16 |
| Lincosamides | Clindamycin | 14 | 21 |
| Rifamycins | Rifampicin | 16 | 20 |
| Streptogramins | Quinupristin | 15 | 19 |
| Nitrofurans | Nitrofurantoin | 14 | 17 |

R: resistant; S: sensitive.
