# Supplementary material for: Prevalence, Virulence Genes, Antimicrobial Susceptibility, and Genetic Diversity of Bacillus cereus Isolated From Pasteurized Milk in China
Source: Front Microbiol. 2018 Mar 26;9:533. doi: 10.3389/fmicb.2018.00533 (PMC5879084; doi:10.3389/fmicb.2018.00533)
Supplement: TABLE S2 — Prevalence of virulence genes in Bacillus cereus isolated from pasteurized milk in China. [file Table_2.docx]

**Supplementary Table 2 Prevalence of virulence genes in *Bacillus cereus* isolated from pasteurized milk in China.**

| Toxigenic genes | Number of strains (%) positive for target gene |
| --- | --- |
| Non-hemolytic enterotoxin genes |  |
| *nheA* | 102 (99%) |
| *nheB* | 102 (99%) |
| *nheC* | 97 (94%) |
| Hemolysin BL genes |  |
| *hblA* | 48 (47%) |
| *hblC* | 70 (68%) |
| *hblD* | 70 (68%) |
| Cytotoxin K gene |  |
| *cytK* | 75 (73%) |
| Potential enterotoxigenic genes |  |
| *hlyⅡ* | 56 (54%) |
| *entFM* | 99 (96%) |
| *bceT* | 77 (75%) |
| Cereulide synthetase gene |  |
| *cesB* | 5 (5%) |
